# Gemcitabine, oxaliplatin and dexamethasone (GemDOx) as salvage therapy for relapsed or refractory diffuse large B-cell lymphoma and peripheral T-cell lymphoma

**DOI:** 10.7150/jca.47031

**Published:** 2021-01-01

**Authors:** Qiu-Dan Shen, Li Wang, Hua-Yuan Zhu, Jin-Hua Liang, Yi Xia, Jia-Zhu Wu, Lei Fan, Jian-Yong Li, Wei Xu

**Affiliations:** 1 Department of Hematology, the First Affiliated Hospital of Nanjing Medical University, Jiangsu Province Hospital, Nanjing 210029, China; Key Laboratory of Hematology of Nanjing Medical University, Nanjing 210029, China; Collaborative Innovation Center for Cancer Personalized Medicine, Nanjing 210029, China.; 2 Pukou CLL Center, Nanjing 210000, China.

**Keywords:** gemcitabine, oxaliplatin and dexamethasone, relapsed or refractory diffuse large B-cell lymphoma, peripheral T-cell lymphoma

## Abstract

**Background** Outcomes of relapsed or refractory diffuse large B-cell lymphoma (DLBCL) and peripheral T-cell lymphoma (PTCL) remain poor. The objective of this study was to evaluate the efficacy and safety of gemcitabine, oxaliplatin and dexamethasone (GemDOx) with or without rituximab as salvage therapy in patients with relapsed or refractory DLBCL and PTCL.

**Materials and Methods:** We retrospectively reviewed patients with relapsed or refractory DLBCL and PTCL receiving GemDOx as salvage therapy between Jul 1, 2011, and Aug 31, 2017.

**Results:** Thirty-three (57.9%) patients with relapsed or refractory DLBCL and 24 (42.1%) with PTCL were included in this study. The median age was 57 years (inter-quartile range 46-67). The overall response rate (ORR) in DLBCL was 48.5% with 27.3% of complete remission (CR), and the 2-year progression-free survival (PFS) and 2-year overall survival (OS) was 21% and 44%. In patients with PTCL, ORR was 50.0% with CR rate of 29.2%; the 2-year PFS and 2-year OS was 28% and 49%, respectively. Common grade 3-4 hematological adverse events were thrombocytopenia (26.3%), anemia (15.7%) and neutropenia (15.7%).

**Conclusion:** With acceptable efficacy and good tolerability, GemDOx might be a new therapeutic option for relapsed or refractory DLBCL and PTCL.

## Introduction

Diffuse large B-cell lymphoma (DLBCL) and peripheral T-cell lymphoma (PTCL) are aggressive lymphomas, accounting for 25-35% and 10-20% of non-Hodgkin lymphomas (NHL), respectively 1-3. The current standard first-line therapy for DLBCL consisting of rituximab plus cyclophosphamide, doxorubicin, vincristine, and prednisone (R-CHOP) has cured approximately 60% of patients with DLBCL 4. More than 30% patients ultimately relapse with 10% present with refractory diseases 4. For patients with PTCL, CHOP-based regimens as commonly used first-line therapy have limited efficacy, with a long-term survival rate of only 10-30% 5, 6. Treatments of relapsed or refractory (R/R) DLBCL and PTCL generally include salvage chemotherapy followed by high-dose therapy and hematopoietic stem cell transplantation (SCT). However, outcomes of these patients are poor. Currently, there are no preferred salvage chemotherapies defined for R/R DLBCL and PTCL. There remains a need to optimize salvage regimens to improve the prognosis of these patients.

Gemcitabine, an analog of cytosine arabinoside, has proven to be effective in the treatment of R/R NHL 7. Gemcitabine-based regimens such as gemcitabine, dexamethasone plus cisplatin (GDP) and gemcitabine plus oxaliplatin (GemOx) have been evaluated in R/R DLBCL and PTCL, with overall response rate (ORR) ranging from 30% to 83% 8-14. It has been reported that platinum derivative oxaliplatin has synergistic effects with gemcitabine in the treatment of NHL, with similar efficacy to cisplatin and reduced renal toxicity 15. To date, very limited data are available on the use of the combination regimen of gemcitabine, oxaliplatin and dexamethasone (GemDOx) in patients with NHL.

Over the last eight years, GemDOx regimen has been employed as a salvage chemotherapy for R/R NHL in our institution. Therefore, we conducted a retrospective study to evaluate the efficacy and safety of GemDOx in patients with R/R DLBCL and PTCL.

## Materials and methods

### Study design and patients

This is a retrospective study in patients with R/R DLBCL and PTCL who received GemDOx with or without rituximab as a salvage therapy. Patients were enrolled from our Hospital from Jul 1, 2011 to Aug 31, 2017. Patient data were collected through reviewing electronic medical records and paper charts.

Patients fulfilling the following criteria were considered for the present study: aged 18 years or older; had histologically confirmed CD20-positive DLBCL, PTCL, not otherwise specified (PTCL, NOS), angioimmunoblastic T-cell lymphoma (AITL) and ALK-negative anaplastic large cell lymphoma (ALCL); had relapsed or refractory disease. Relapse was defined as progressive disease after achieving complete remission (CR) by first-line or later-line therapy. Refractory was defined as progressive disease after at least four cycles of first-line therapy or stable disease as best response after at least two cycles of later-line therapy or relapse shorter than 12 months after autologous stem cell transplantation (ASCT). Patients with incomplete medical data or lost to follow-up were excluded from this study.

### Treatment

The GemDOx regimen was administered as follows: gemcitabine 1 g/m^2^ intravenously on day 1 and day 5; oxaliplatin 75 mg/m^2^ intravenously on day 1; and dexamethason 40 mg intravenously on day 1-4. Patients with DLBCL also received rituximab at a dose of 375 mg/m^2^ intravenously on day 0 of each cycle in the event that they relapsed longer than 6 months after previous rituximab containing treatment. The cycle was repeated every 21 days. Number of cycles was determined by response and up to six cycles were administered.

### Response assessment

Patients' responses were assessed every two cycles by the treating physician according to the Revised Response Criteria for Malignant Lymphoma 16. Fluoro deoxyglucose-positron emission tomography (FDG-PET) was not routinely performed for the assessment of response to GemDOx. Patients were regularly followed up every three to six months.

Covariates including International Prognostic Index (IPI) score, stage of disease, ECOG performance status, extranodal sites, bone marrow involvement (BMI), bulky disease, B symptoms, lactate dehydrogenase (LDH) and β2-microglobulin (β2-MG) were determined at diagnosis. The germinal center B-cell like (GCB) or non-germinal center B-cell like (non-GCB) subtype was identified at diagnosis on immunohistochemistry of paraffin-embedded tissue based on Hans's algorithm. The line of therapy and relapse or refractory status was determined before the commencement of GemDOx.

### Statistical analysis

Characteristics of patients were summarized using descriptive statistical methods. ORR in different subgroups was compared using χ^2^ tests or Fisher exact tests. PFS was defined as the time from the commencement of GemDOx to disease progression, death from any cause, or the date of the last follow-up visit, whichever occurred first. OS was measured from the time of the commencement of GemDOx to death from any cause or the date of the last follow-up visit, whichever occurred first. PFS and OS were estimated by use of Kaplan-Meier survival curves. The log-rank test was used to compare variables of interest. A two-sided *P* value less than 0.05 was considered significant. Statistical analyses were done using SPSS version 21.

## Results

### Patients' characteristics

**Figure 1:**
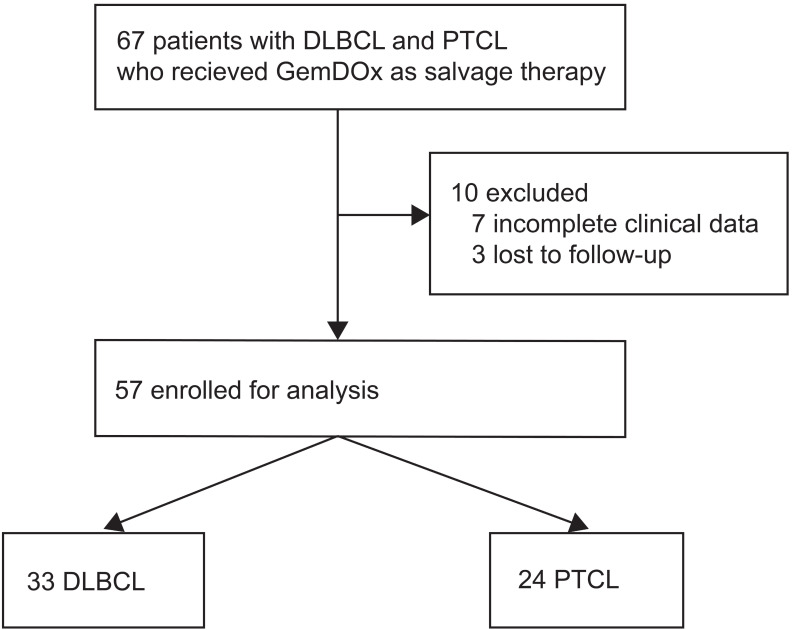
Flowchart of eligibility.

Between Jul 1, 2011 and Aug 31, 2017, 67 patients who received GemDOx as a salvage therapy were identified, and 57 were eligible for the analysis including 33 (57.9%) with DLBCL and 24 (42.1%) with PTCL (**Figure 1**). The characteristics of patients at diagnosis are summarized in **Table 1.** The median age of patients was 57 years (inter-quartile range 46-67). Most patients were presented with Ann Arbor stage III-IV and had ECOG performance status score of 0 or 1. In the cohort of patients with DLBCL, 14 (42.4%) patients had an IPI score of 3 or higher. Twenty-four (72.5%) were identified as non-GCB subtype. In the PTCL cohort, 5 (20.8%) patients had an IPI score of 3 or higher. Subtypes of PTCL included PTCL-NOS (13 [54.2%]), AITL (8 [33.3%]), and ALK-negative ALCL (3 [12.5%]).

Initial therapy and characteristics of patients at the time of GemDOx in both cohorts are summarized in **Table 2.** Patients received CHOP, dose adjusted etoposide, prednisone, vincristine, cyclophosphamide and doxorubicin (EPOCH) or cyclophosphamide, doxorubicin, vincristine, prednisone and etoposide (CHOPE) as first-line chemotherapy. In the DLBCL cohort, 26 (78.8%) patients received rituximab in the first-line therapy. Twenty-one (63.6%) patients with DLBCL were at first relapse, 3 (9.1%) were in second or multiple relapse and 9 (27.3%) had refractory diseases. Early relapse, which is defined as relapse within 12 months to first-line therapy, was in 16 of 21 (76.1%) patients with relapsed DLBCL. In the PTCL cohort, 15 (62.5%) patients were at first relapse, with 10 (73.3%) of 15 patients at early relapse. Remaining patients (9 [37.5%] of 24) with PTCL had refractory diseases.

### Efficacy

Overall, 160 cycles of GemDOx chemotherapy was administered. Among the total of 57 patients, 33 (57.9%) patients received 1-2 cycles, 11 (19.2%) received 3 cycles and 13 received ≥4 cycles of GemDOx. The median number of cycles was 2 (range 2-6) per person. Fifteen (45.5%) patients with DLBCL received rituximab combined with GemDOx. The response at the end of GemDOx treatment is shown in **Table 3.** Sixteen of 33 (48.5%) patients with DLBCL had the response, with 9 (27.3%) achieving CR or CRu. The overall response rate did not differ significantly in different subgroups according to age, sex, disease status (relapsed or refractory), Ann Arbor stage, IPI, cell of origin subtype or whether rituximab was used. In the cohort of PTCL, 12 (50.0%) patients achieved ORR, with 7 (29.2%) achieving CR or CRu. Of note, patients with relapsed PTCL had a high ORR of 66.7% with an ORR of 22.2% in those with refractory diseases, but the difference was not statistically significant (*P*=0.09). The ORR in other subgroups of patients with PTCL did not differ significantly according to age, sex, Ann Arbor stage, IPI or histological subtypes. Overall 6 of 28 (21.4%) transplant-eligible patients proceeded to SCT.

**Figure 2:**
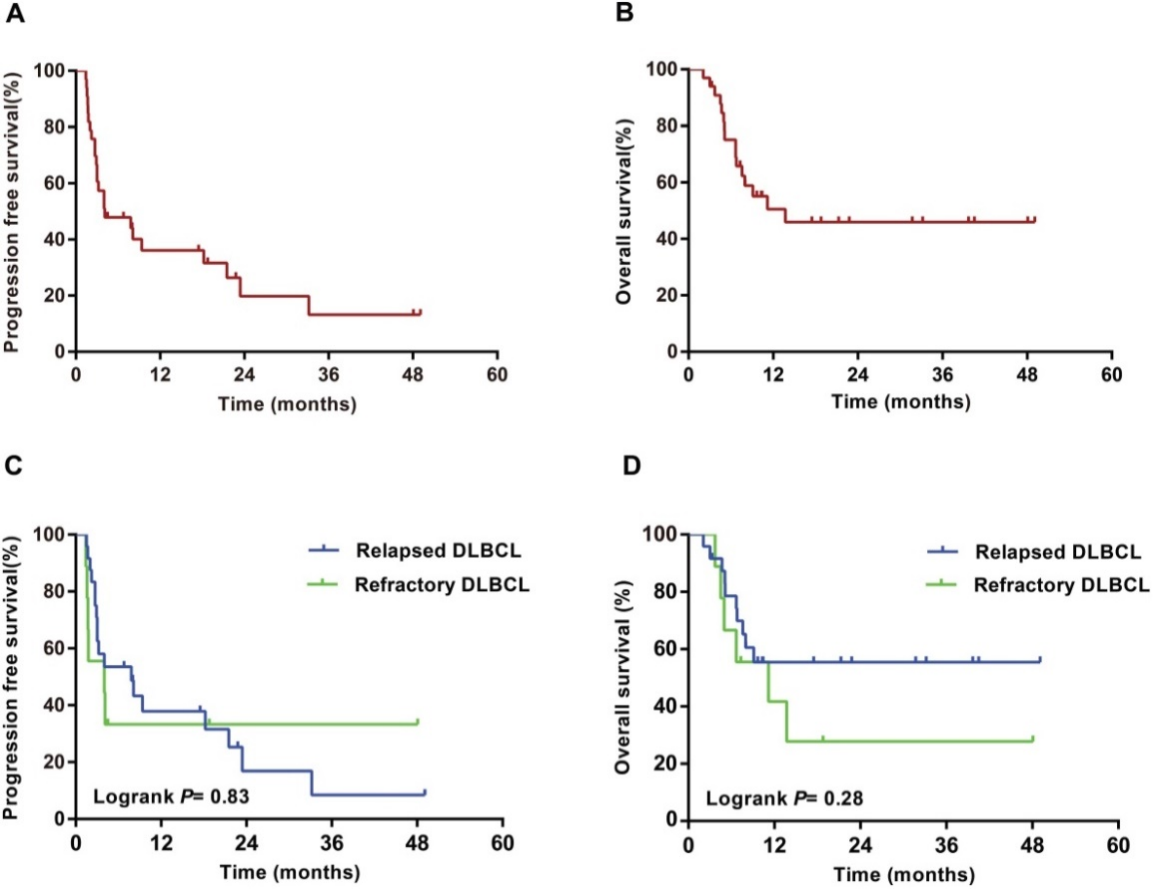
** Progression-free survival and overall survival of patients with DLBCL from the commencement of GemDOx.** (**A**) Progression-free survival of entire DLBCL cohort. (**B**) Overall survival of entire DLBCL cohort. (**C**) Progression-free survival of patients with relapsed DLBCL versus refractory DLBCL. (**D**) Overall survival of patients with relapsed DLBCL versus refractory DLBCL.

**Figure 3:**
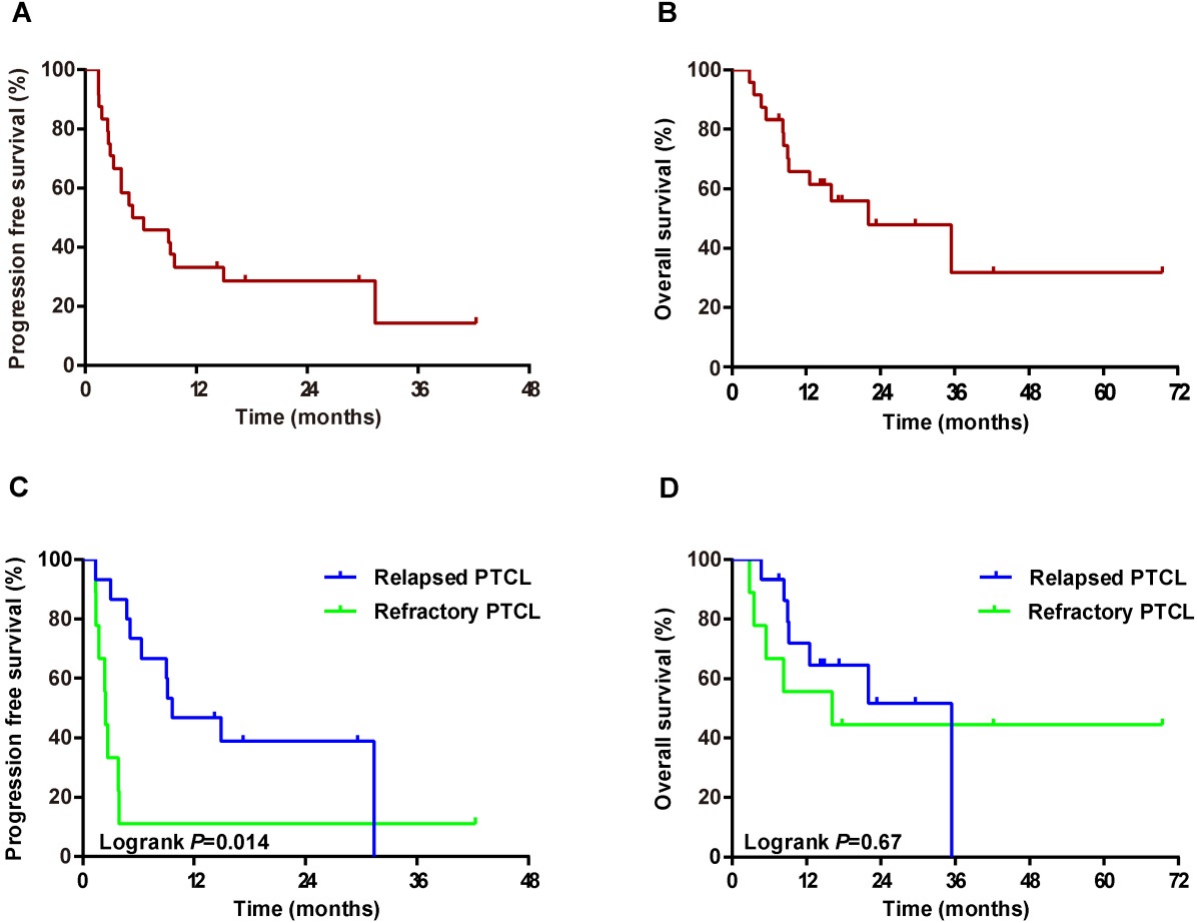
** Progression-free survival and overall survival of patients with PTCL from the commencement of GemDOx.** (**A**) Progression-free survival of entire PTCL cohort. (**B**) Overall survival of entire PTCL cohort. (**C**) Progression-free survival of patients with relapsed PTCL versus refractory PTCL. (**D**) Overall survival of patients with relapsed PTCL versus refractory PTCL.

With a median follow-up of 21 months (range 2-70) from the start of GemDOx treatment, 16 patients with DLBCL died (1 from severe pneumonia, 15 from lymphoma), and 12 with PTCL died (1 from acute cerebral infarction, 11 from lymphoma). In the DLBCL cohort, median PFS was 4 months (95%CI 0-10), and median OS was 14 months (95%CI not reached). 1-year PFS and OS were 35% (95%CI 17-53) and 49% (31-67); 2-year PFS and OS were 21% (5-37) and 44% (24-64), respectively. PFS (*P*=0.83) and OS (*P*=0.28) did not differ significantly between patients with relapsed DLBCL and those with refractory DLBCL (**Figure 2**). In the PTCL cohort, median PFS was 5 months (95%CI 0-11) and median OS was 22 months (3-40). 1-year PFS and OS were 33% (95%CI 13-53) and 66% (46-86); 2-year PFS and OS were 28% (10-46) and 49% (27-71), respectively. PFS was significantly longer in patients with relapsed PTCL than those with refractory PTCL (*P*=0.014), while OS did not differ significantly between the two subgroups (*P*=0.67) (**Figure 3**).

### Safety

Because of the retrospective design of this study, some records about detailed or delayed toxicities were incomplete. The most frequent treatment-related adverse events (AEs) recorded in this study were hematological toxicities, occurring in 29 (50.9%) of 57 patients. Non-hematological AEs were documented in 11 (19.3%) patients and all were mild, including gastrointestinal complications, elevation of aminotransferases and atrial fibrillation. Dose reductions were recorded in two patients older than 60 years old. One was due to severe hematologic toxicities in the previous treatment, with a 20% reduction in the dose of gemcitabine and oxaliplatin during the second cycle. The other frail patient received 10% dose reduction of all the three drugs during the treatment. No patient discontinued GemDOx treatment because of AEs. No treatment-related death was documented.

## Discussion

Relapsed or refractory aggressive lymphomas remain a therapeutic challenge with poor outcomes and rare long-term survivors. Previous studies have evaluated the efficacy of major salvage therapies in R/R NHL 17, 18. One randomized trial (CORAL study) in R/R DLBCL showed an ORR of 63% after rituximab plus ifosfamide, carboplatin, etoposide (R-ICE) and 64% after rituximab plus cisplatin, cytarabine, dexamethasone (R-DHAP) 17. The 3-year PFS was 37% (95% CI 31-42) and 3-year OS was 49% (43-55), with no difference between R-ICE and R-DHAP 17. In another randomized study (LY.12 study) of patients with R/R NHL, most of them with DLBCL, the ORR with GDP was 45.1% (13.5% CR) and with DHAP was 44.0% (14.3% CR) 18. The 4-year event free survival (EFS) rate was 43% (95%CI 34-51) with GDP and 48% (39-57) with DHAP; the 3-year OS rate was 62% (53-69) and 63% (54-71), respectively 18. GDP shows a non-inferior efficacy and less toxicity compared with DHAP. Several other studies have also confirmed the efficacy of GDP in the treatment of R/R DLBCL with ORRs of approximately 49% 8, 10.

In our study, based on the safety and synergistic activity of gemcitabine and oxaliplatin, we modified the GDP regimen by replacing cisplatin with oxaliplatin. We found an encouraging efficacy of GemDOx in R/R DLBCL with an ORR of 48.5% (27.3% CR/CRu) and 2-year PFS and 2-year OS of 21% (95%CI 5-37) and 44% (24-64), respectively. The response rate we observed is comparable to previously reported with GDP. Nonetheless, the overall survival appears less satisfactory compared with the aforementioned two randomized studies. Previous studies have found that early relapse and prior rituximab treatment are associated with poor outcomes in R/R DLBCL 17. In our study, 76% of patients experienced early relapse or had refractory diseases, which was higher than the two randomized studies. More patients (78.8%) received rituximab as first-line treatment. In a population-based study of 152 patients with R/R DLBCL who received GDP as salvage therapy, the 2-year PFS and OS were 21% and 28% 10, which appears lower than that we observed with GemDOx. In clinical practice, there might be more patients with poor clinical characteristics than in randomized trials. In addition, GemDOx showed a similar efficacy regardless of patients' baseline characteristics such as age, sex, Ann Arbor stage, IPI, cell of origin subtype, disease status or whether rituximab used or not. This indicates that GemDOx might have abroad activity in the treatment of R/R DLBCL. However, the hypothesis requires further investigations.

Outcomes of R/R PTCL are inferior to DLBCL. Gemcitabine has been reported to have better efficacy in R/R PTCL, with a single-agent response rate of 51% 19. Among gemcitabine-based regimens, GDP has emerged as an effective chemotherapy in the treatment of R/R PTCL. The ORR of GDP ranges from 30% to 72% in previous studies 12, 13, 18. In this study, GemDOx achieved an ORR of 50.0%, and 2-year PFS and OS were 28% (10-46) and 49% (27-71), respectively. Notably, we found a high ORR of 66.7% and longer PFS in patients with relapsed PTCL compared with those with refractory diseases.

To date, only a few studies have evaluated the combination regimen of GemDOx, each of which using different strategies of administration and with limited smaple size 20, 21. In a study of 24 elderly patients with refractory or relapsed PTCL, GemDOx was given at the dose of gemcitabine 1 g/m^2^ on day 1, oxaliplatin 100 mg/m^2^ on day 1, and dexamethasone 20 mg on day 1-4, and showed an unsatisfactory ORR of 25% 20. In our study, with an escalated dose of gemcitabine and dexamethasone, we observed a higher ORR than the previous study. In another phase II study of 29 patients with R/R aggressive NHL, GemDOx was administered as follows: gemcitabine 1 g/m^2^ on day 1 and day 15, oxaliplatin 85 mg/m^2^ on day 1 and day 15, and dexamethasone 40 mg on day 1-4 21. This study resulted in an ORR of 47.1% with CR of 23.5%. It appears that the biweekly dose-dense administration of gemcitabine and oxaliplatin failed to improve the response of patients 21. However, it is difficult to make direct comparisons among these studies because of limited data available, heterogeneous subtypes and different patient populations included.

In our study, we also adjusted the dosing interval of gemcitabine, which was given in Day 1 and Day 5 to shorten the hospitalization time. Although gemcitabine was given in a short interval of five days, the safety profile of GemDOx was favorable. Hematological AEs were moderate and manageable. A relatively high incidence of thrombocytopenia was documented in around one quarter of patients, which might be resulted from gemcitabine. Non-hematological AEs were also mild and reversible.

Limited by the small sample size and short follow-up time, this study was not powered enough to show differences in response and survival among different patient categories. As high-dose therapy followed by SCT remains the backbone treatment of R/R NHL 22, we could not assess the effect of GemDOx on SCT in this study due to small numbers of patients proceeding to SCT. Further investigations are required to confirm the findings of this study.

Recently, a list of novel agents, including the tyrosine kinase inhibitors targeting the B-cell receptor signaling pathway, immunomodulators, histone deacetylase inhibitors and immunotherapies, have shown promising efficacy in the treatment of NHL 23, 24. Integration of the new agents into the established chemotherapy may have potential to improve the outcomes of patients with R/R aggressive lymphomas. Therefore, we have initiated a phase I study to investigate the safety and efficacy of the combination therapy of lenalidomide, rituximab and GemDOx (R2-GemDOx) in R/R DLBCL (ClinicalTrials.gov, number NCT03795571).

In conclusion, the results of our study suggest that with acceptable efficacy and good tolerability, GemDOx might be a new therapeutic option for R/R DLBCL and PTCL. Further investigations on combinations of GemDOx with novel agents are being evaluated in the treatment of R/R aggressive lymphomas.

## Figures and Tables

**Table 1:** Patients' characteristics at diagnosis

Characteristics	DLBCL (n=33)	PTCL (n=24)
Median age, years (IQR)	57 (44-63)	57 (49-62)
**Sex**		
Men	20 (60.6)	16 (66.6)
Women	13 (39.4)	8 (33.3)
***Histological subtype***		
**Diffuse large B-cell lymphoma**		
Germinal centre B-like	9 (27.3)	-
Non-germinal centre B-like	24 (72.5)	-
**Peripheral T cell lymphoma**		
Peripheral T cell lymphoma, not other specified	-	13 (54.2)
Angioimmunoblast lymphoma	-	8 (33.3)
Anaplastic large cell lymphoma	-	3 (12.5)
ECOG performance status score ≥2	6 (18.2)	4 (16.7)
**Ann Arbor stage**		
I-II	4 (12.1)	4 (16.7)
III-IV	29 (87.9)	20 (83.3)
Lactate dehydrogenase >ULN	26 (78.8)	12 (50.0)
β2-microglobulin >2.53 mg/L	22/31 (71.0)	16/21 (76.2)
Extranodal sites ≥2	6 (18.2)	2 (8.3)
Bone marrow involvement	4 (12.1)	6 (25.0)
Bulky disease ≥7.5 cm	5 (15.2)	0 (0)
B symptom	14 (42.4)	14 (58.3)
**IPI score**		
0 or 1	7 (21.2)	10 (41.7)
2	12 (36.4)	9 (37.5)
3	8 (24.2)	1 (4.2)
4 or 5	6 (18.2)	4 (16.7)

IQR, Inter-quartile range; ECOG, Eastern Cooperative Oncology Group; ULN, upper limit of normal; IPI, International prognostic index.

**Table 2:** Initial therapy and characteristics of patients at the time of GemDOx

	DLBCL, n (%) (n=33)	PTCL, n (%) (n=24)
**First-line chemotherapy**		
R-CHOP	12 (36.4)	-
R-DA-EPOCH	14 (42.4)	-
CHOP	4 (12.1)	6 (25.0)
DA-EPOCH	3 (9.1)	14 (58.3)
CHOPE	-	4 (16.7)
**Disease status**		
First relapse	21 (63.6)	15 (62.5)
Second or multiple relapse	3 (9.1)	0 (0)
Primary refractory	5 (15.2)	8 (33.3)
Refractory to second-line or later-line therapy	4 (12.1)	1 (4.2)
Median no. of previous treatment regimens (range)	1 (1-4)	1 (1-3)
Median time from initial diagnosis to GemDOx, months (IQR)	8.1 (4.8-15.2)	7.2 (2.0-17.2)
Median time from last treatment to GemDOx, months (IQR)	1.3 (1.0-6.8)	1.8 (0.9-6.3)
Prior ASCT	3 (9.1)	2 (8.3)
**Duration of response to last treatment**		
≥ 1 year	3 (9.1)	4 (16.7)
< 1 year	30 (90.9)	20 (83.3)

R, rituximab; CHOP, cyclophosphamide, doxorubicin, vincristine, and prednisone; DA-EPOCH, dose-adjusted etoposide, prednisone, vincristine, cyclophosphamide and doxorubicin; CHOPE, cyclophosphamide, doxorubicin, vincristine, prednisone and etoposide; IQR, Inter-quartile range; ASCT, autologous stem cell transplantation.

**Table 3:** Responses to GemDOx treatment according to patients' characteristics

Characteristics	DLBCL, n (%)						PTCL, n (%)					
	CR/CRu	PR	SD	PD	ORR	*P*	CR/CRu	PR	SD	PD	ORR	*P*
All patients	9 (27.3)	7 (21.2)	11 (33.3)	6 (18.2)	16 (48.5)	-	7 (29.2)	5 (20.8)	7 (29.2)	5 (20.8)	12 (50.0)	-
***Histological subtype***												
**DLBCL**												
GCB	3 (33.3)	2 (22.2)	3 (33.3)	1 (11.1)	5 (55.6)	0.71	-	-	-	-	-	-
Non-GCB	6 (25.0)	5 (20.8)	8(33.3)	5 (20.8)	11 (45.8)		-	-	-	-	-	-
**PTCL**												
PTCL, NOS	-	-	-	-	-	-	2 (15.4)	4 (30.8)	3 (23.1)	4 (30.8)	6 (46.2)	0.82
AITL	-	-	-	-	-	-	3 (37.5)	1 (12.5)	3 (37.5)	1 (12.5)	4 (50.0)	
ALCL	-	-	-	-	-	-	2 (66.7)	0	1 (33.3)	0	2 (66.7)	
**Disease status**												
Relapsed	9 (37.5)	4 (16.7)	7 (29.2)	4 (16.7)	13 (54.2)	0.42	6 (40.0)	4 (26.7)	4 (26.7)	1 (6.7)	10 (66.7)	0.09
Refractory	0	3 (33.3)	4 (44.4)	2 (22.2)	3 (33.3)		1 (11.1)	1 (11.1)	3 (33.3)	4 (44.4)	2 (22.2)	
**Sex**												
Male	5 (25.0)	5 (25.0)	7 (35.0)	3 (15.0)	10 (50.0)		5 (31.3)	3 (18.8)	4 (25.0)	4 (25.0)	8 (50.0)	1.00
Female	4 (30.8)	2 (15.4)	4 (30.8)	3 (23.1)	6 (46.2)	1.00	2 (25.0)	2 (25.0)	3 (37.5)	1 (12.5.0)	4 (50.0)	
**Age**												
>60 years	5 (38.5)	1 (7.7)	4 (30.8)	3 (23.1)	6 (46.2)	1.00	3 (50.0)	0	2 (33.3)	1 (16.7)	3 (50.0)	1.00
≤60 years	4 (20.0)	6 (30.0)	7 (35.0)	3 (15.0)	10 (50.0)		4 (22.2)	5 (27.8)	5 (27.8)	4 (22.2)	9 (50.0)	
**Ann Arbor stage**												
I-II	2 (50.0)	1 (25.0)	0	1 (25.0)	3 (75.0)	0.34	3 (75.0)	0	1 (25.0)	0	3 (75.0)	0.59
III-IV	7 (24.1)	6 (20.7)	11 (37.9)	5 (17.2)	13 (44.8)		4 (20.0)	5 (25.0)	6 (30.0)	5 (25.0)	9 (45.0)	
**IPI score**												
0-2	6 (31.6)	5 (26.3)	5 (26.3)	3 (15.8)	11 (57.9)	0.30	7 (36.8)	4 (21.1)	4 (21.1)	4 (21.1)	11 (57.9)	0.32
3-5	3 (21.4)	2 (14.3)	6 (42.9)	3 (21.4)	5 (35.7)		0	1 (20.0)	3 (60.0)	1 (20.0)	1 (20.0)	

DLBCL, diffuse large B-cell lymphoma; GCB, germinal centre B; PTCL, NOS, peripheral T cell lymphoma, not other specified; AITL, angioimmunoblast lymphoma; ALCL, anaplastic large cell lymphoma; CR, complete remission; CRu, complete remission/unconfirmed; PR, partial remission; SD, stable disease; PD, progressive disease; ORR, overall response rate.
